# The Healthy Migrant Families Initiative: development of a culturally competent obesity prevention intervention for African migrants

**DOI:** 10.1186/s12889-015-1628-2

**Published:** 2015-03-19

**Authors:** Andre MN Renzaho, Jennifer A Halliday, David Mellor, Julie Green

**Affiliations:** School of Social Sciences and Psychology, University of Western Sydney, Locked Bag 1797, Penrith, 2751 NSW Australia; School of Psychology, Faculty of Health, Deakin University, 221 Burwood Hwy, Burwood, VIC 3125 Australia; Raising Children Network, Parenting Research Centre Level 5, 232 Victoria Parade, East Melbourne, VIC 3002 Australia; Murdoch Childrens Research Institute, Flemington Road, Parkville, VIC 3052 Australia; Department of Paediatrics, The University of Melbourne, Flemington Road, Parkville, VIC 3052 Australia

**Keywords:** Obesity prevention, African migrants, Intervention, Cultural competence, Healthy lifestyle, Parenting

## Abstract

**Background:**

Although obesity among immigrants remains an important area of study given the increasing migrant population in Australia and other developed countries, research on factors amenable to intervention is sparse. The aim of the study was to develop a culturally-competent obesity prevention program for sub-Saharan African (SSA) families with children aged 12–17 years using a community-partnered participatory approach.

**Methods:**

A community-partnered participatory approach that allowed the intervention to be developed in collaborative partnership with communities was used. Three pilot studies were carried out in 2008 and 2009 which included focus groups, interviews, and workshops with SSA parents, teenagers and health professionals, and emerging themes were used to inform the intervention content. A cultural competence framework containing 10 strategies was developed to inform the development of the program. Using findings from our scoping research, together with community consultations through the African Review Panel, a draft program outline (skeleton) was developed and presented in two separate community forums with SSA community members and health professionals working with SSA communities in Melbourne.

**Results:**

The ‘Healthy Migrant Families Initiative (HMFI): Challenges and Choices’ program was developed and designed to assist African families in their transition to life in a new country. The program consists of nine sessions, each approximately 1 1/2 hours in length, which are divided into two modules based on the topic. The first module ‘Healthy lifestyles in a new culture’ (5 sessions) focuses on healthy eating, active living and healthy body weight. The second module ‘Healthy families in a new culture’ (4 sessions) focuses on parenting, communication and problem solving. The sessions are designed for a group setting (6–12 people per group), as many of the program activities are discussion-based, supported by session materials and program resources.

**Conclusion:**

Strong partnerships and participation by SSA migrant communities enabled the design of a culturally competent and evidence-based intervention that addresses obesity prevention through a focus on healthy lifestyles and healthy families. Program implementation and evaluation will further inform obesity prevention interventions for ethnic minorities and disadvantaged communities.

## Background

The United Nations’ Food and Agriculture Organisation reported that, at 26.8%, sub-Saharan Africa has the highest prevalence of undernourishment, representing 234 million individuals who suffered from hunger in the 2010–2012 period and a growth of 64 million in the last 2 decades [1]. However, upon migration to industrialised countries, sub-Saharan African migrants are at increased risk of obesity and obesity-related diseases [2-5]. Numerous factors have been associated with the drastic increase in migrants’ body mass index (BMI), one of which is acculturation [6].

Acculturation refers to the phenomenon through which migrant populations adopt the cultural patterns (e.g., values, behaviours, norms, attitudes and beliefs) of their host society whilst perhaps maintaining aspects of their own culture to varying degrees [7]. In fact, emerging evidence suggests that there is an association between acculturation and overweight and obesity among migrant children and adults from low-moderate income countries to industrialised countries [6,8-11]. However, the prevalence of overweight and obesity is significantly lower among migrants who maintain some elements of their traditional cultural practices [6,12,13].

Although obesity remains an important area of study given the increasing migrant population in Australia and other developed countries, research on factors amenable to intervention is sparse. Most importantly, sub-Saharan African migrants are under-represented in obesity related research programs due to their low health literacy, poor English proficiency, and other cultural barriers. Studies seeking to determine socio-cultural factors to be included in obesity prevention programs targeting marginalised migrant communities are urgently needed. Therefore, the aim of this study was to develop a culturally-competent obesity prevention program for sub-Saharan African families with children aged 12–17 years using a community-partnered participatory approach.

## Methods

### Program development theory

The cultural competence framework was used to guide the development of the program. Cultural competence refers to “a set of congruent behaviours, attitudes and policies that come together in a system, agency or among professionals and enable that system, agency or those professions to work effectively in cross-cultural situations” [14]. Cultural competence is not just about awareness of cultural differences, but the capacity of the system to improve health through the integration of culture into program delivery [15]. Therefore, cultural competence in health and research refers to the ability of researchers and health practitioners to take into account the culture and diversity of the target population when developing a health promotion program, from the conception of the research ideas to the conduct of the research and the dissemination and application of the research findings. In this sense, cultural competence offers both a conceptual and practical approach to developing a culturally-competent health promotion program.

Sawyer and colleagues [16] have identified three strategies to achieve cultural competence in research: cultural knowledge, sensitivity, and collaboration. Gibbs and colleagues go further, proposing nine complementary strategies to achieve cultural competence in research [17] focusing on stages of research implementation: 1) forming partnership, 2) defining the research questions, 3) identifying data sources and target populations, 4) appointing staff, 5) recruiting participants, 6) data collection, 7) data analysis and evaluation, 8) reporting and disseminating, and 9) using the findings to develop an intervention. At each research stage, the authors provide examples of culturally competent practices that maximise participation among ethnic minorities and disadvantaged communities. However, there are some similarities and overlaps in these cultural competence models in terms of contents and application. We integrated and synthesised the different models to develop a cultural competence framework comprising 10 strategies [17], which informed the development of the program (Table 1). The development of the intervention used a trans-generational psycho-educational approach to obesity related behaviours. It included education sessions focused on healthy lifestyles in a new environment, and it addressed the intergenerational acculturation gap through a focus on parenting, communication and problem solving within the family.

**Table 1:** **Cultural competence strategies informing program development**

**Strategies**	**Our approach**
**1. Defining research questions: Cultural knowledge**	
• Does the question reflect an authentic awareness of the culture?	• Our previous work in this population and pilot studies have established evidence on the current health problems prevalent in this population
• Has previous research related to the population been critically acknowledged?	• Follow up studies will be conducted to establish the extent of the problem
• How does the question relate to current health care problems?	• African Review Panel ensured that the methodology is culturally appropriate
• Is there an indication of knowledge of appropriate ways of developing and maintaining trust?	
• Is the guiding methodology consistent with culture?	
**2. Cultural sensitivity**	
• What mechanisms are used to enhance cultural appropriateness?	• A cultural competence framework and community-partnered participatory approach was applied
• Do the participants define the health issue as a priority problem?	• African Review Panel and African bilingual workers were integral in advising about cultural appropriateness
• Is there evidence of a flexible process throughout data collection?	• Our previous research indicated this to be a priority problem for this sub-population
**3. Collaboration**	
• Was the problem developed with the target population?	• Community consultation and collaboration occurred throughout the process – from the pilot studies to the development of the final HMFI program (refer to main text)
• At what level were the participants involved?	• The level of participation varied depending on the phase of the project – for instance, the ANGELO workshop was community driven, the pilot interviews were conducted by community members, and the community were consulted at numerous stages of the HMFI program development
• Were participants involved in the development and implementation of methodology?	
**4. Forming partnerships**	
• Did the researchers work through gatekeepers to establish peer educators (community workers who are of the same cultural background as participants matched to the culture, language, gender, age and life stage of the research participants)?	• Formation of partnerships with community gatekeepers was essential in collecting pilot data – often the person collecting the data was required to seek permission from the President (gatekeeper) of the community before commencing work within the community group. The same will be vital in implementing the program.
**5. Identifying data sources and target populations**	
• Do the researchers recognize their own cultural framework and its influence on the research approach?	• African review Panel and African bilingual workers ensured that a culturally congruent approach was maintained
**6. Appointing staff**	
• Did the researchers ensure involvement of peers, not just community gatekeepers?	• The African Review Panel and African bilingual workers are both composed of a range of people – some are community gatekeepers, some are community-nominated, and some are involved for other reasons e.g. they have work experience or a personal interest
**7. Recruitment of sample**	
• Do the researchers recognize diversity within cultural groups?	• The African Review Panel and African bilingual workers are key in assisting the researchers in understanding the cultural diversities within the African communities
• Recognize potential power imbalances in working and consultation?	• To minimize power-balance effects, participant recruitment utilized a strategy where communities elected one of their own for training in recruiting families to the study and conducting the interviews.
• Recognize differences in defining language and cultural identity?	
**8. Data collection**	
• Is the methodology responsive to cultural and migration considerations (e.g. family groups rather than individual interviews, use of professional interpreters rather than family members)	• The methodology was culturally appropriate due to input from the African Review Panel
• Were data collected by local community organizations or community members?	• Data for the pilot studies were collected by community elected members who received training in data collection methods (e.g. interview techniques, ethical considerations)
**9. Analysis/evaluation**	
• Were peer educators involved in the analysis and interpretation of the data?	• Two community forums were held to present and discuss the results of the pilot studies and discuss the plans for the program
• Was there feedback from the participants to confirm the results?	• Feedback was also sought from the African Review Panel into the content of the program
**10. Reporting/disseminating findings**	
• Was there an opportunity for the community to discuss findings and generate solutions?	• In addition to the community forums and African Review Panel meetings (see box above), the ANGELO process (on which one of the pilot studies was based) [25] allowed the community to generate their own solutions, which fed back into the HMFI program plan

### Community participatory approach

An interactive community-partnered participatory approach was adopted, whereby the intervention was developed in collaborative partnership with communities, and community members were recruited and trained to participate in all aspects of the project implementation [18,19]. Effective engagement with the community of interest is critical, both to maximise the relevance and acceptance of the developed program [18,19]. This participatory approach was sustained through the mobilization of the African Review Panel, established as part of the African Migrant Capacity Building and Performance Appraisal framework [20-24]. Panel members were either nominated by their respective communities or recruited for their knowledge and expertise through community health workers and African community organisations’ networks. As in previous work, the panel acted as the study’s community-based steering committee as well as overseeing community mobilization and recruitment for the pilot studies, the scoping projects that informed the intervention [20-24] and the intervention components’ mapping study [25]. The panel also assisted the research team in interpretation and dissemination of the pilot and mapping study results within the community, and participated in the development and revision of the program outline, sessions and materials. Recently, the African Review Panel has been expanded to form part of the Child Obesity Prevention Advisory Council to assist in assessing the community readiness to engage with obesity prevention interventions in a program of research funded by the Australian government.

### Target population and the program development process

The target population was families with children aged 12–17 years (see proposed mode of implementation below). Our previous research in this subpopulation shows that sub-Saharan African children and adolescents are at increased risk of obesity, but factors predisposing them to obesity are not yet well understood [6,20-27]. The project development was therefore designed to extend the evidence base through three phases. In Phase 1, we sought to qualitatively describe obesity, its risk factors, and how to improve the health of African families in Melbourne. Three pilot studies were carried out during 2008 and 2009 which included focus groups, interviews, and workshops with African parents, teenagers and health professionals [21,24]. Analyses revealed that the main drivers of obesity among sub-Saharan African children were related to intergenerational differences in parenting beliefs, family functioning, health literacy and lifestyle in a new culture.

In the second phase, a survey was conducted among African teenagers and their parents living in Melbourne to quantify various aspects of their health (including BMI, acculturation, food habits, parenting styles, and family functioning). The first survey was exploratory in nature and sought to test the recruitment approaches, to evaluate the application of parenting and family functioning scales, and to identify aspects of parenting and family functioning associated with obesity [23]. The study involved a total of 208 participants (104 parents and 104 offspring). Obesity emerged as a significant health issue; inconsistent discipline and lack of parental supervision were responsible for a significant proportion of variance in children’s BMI, which supported findings in Phase 1. The African Review Panel facilitated dissemination of these findings to the community, and four workshops were subsequently conducted to identify factors amenable to intervention. Using the Analysis Grid for Elements Linked to Obesity (ANGELO) framework [28], community members prioritized health behaviours, skill and knowledge gaps, and environments for change to be included in family-based childhood obesity prevention programs. The family priorities fell into four categories: behaviours for change; health knowledge gaps; health literacy and skills for better health; and home/family environments [25]. Results from the ANGELO workshops were helpful for indicating the types of strategies that might be helpful for the community and also highlighted low health literacy, particularly amongst parents, which was a barrier to health promotion and information-seeking for the community.

Finally, the development of the program outline and program materials involved a number of steps. Informed by findings from Phases 1 and 2, literature reviews, and community consultations through the African Review Panel, a draft program outline was developed and presented in two separate community forums with African community members and health professionals working with African communities in Melbourne. The first forum was conducted at a community event in December 2011 to celebrate the African Review Panel’s contribution to the project, disseminate findings from Phases 1 and 2 and gather feedback on the draft program outline. The second forum, in March 2012, provided other African community members with an opportunity to hear the program results to date and provide feedback on the draft program plan with the assistance of bilingual workers. Both forums were effective in obtaining useful feedback that informed additional community consultation and finalisation of the final program, ‘The Healthy Migrant Families Initiative: Challenges and Choices’ (HMFI).

Education materials to accompany the program were developed and informed by academic and grey literature, consultations with parenting experts, the research team, nutrition, and health professionals working with African families, the African Review Panel, and health providers to the African communities in Melbourne.

Ethical approvals for all phases of the project were obtained from Deakin University Human Research Ethics Committee and/or Monash University Human Research Ethics Committee.

## Results

### Program overview

The Healthy Migrant Families Initiative: Challenges and Choices program was designed to assist African families in their transition to life in a new country, by providing them with culturally sensitive and relevant training about healthy eating, active living, and communication and problem solving within the family unit. The goal of the intervention was to promote healthy eating and physical activity levels among children by strengthening family cohesion, enhancing family resilience, and promoting whole family lifestyle change. The program content consists of nine sessions, each approximately 1½ hours in length, which are divided into two modules based on their topic. The first module ‘Healthy lifestyles in a new culture’ (5 sessions) focuses mainly upon healthy eating, but also touches on active living and healthy body weight. The second module ‘Healthy families in a new culture’ (4 sessions) focuses on parenting, communication and problem solving. The session titles, purposes and key messages, and activities belonging to each module are presented in Table 2.

**Table 2:** **The components of the ‘Healthy Migrant Families Initiative: Challenges and Choices’**

**Session number**	**Module**	**Session title**	**Purpose**	**Key messages**	**Activities**
1	Healthy families in a new culture	Living in Australia and how it affects your health	To identify and discuss the differences between living in Australia and living in Africa	• Life in Africa and life in Australia are different in many ways (culture, lifestyle, food, transport, school, support, etc.), which all impact on the health	Group discussion and activities to introduce the parents to each other and some of the basic concepts to be covered during the program:
					• Introductions/icebreakers
					• Defining health (ecological definition)
					• Devising strategies for living a healthy life
					• The differences between living in Africa and Australia
2	Healthy families in a new culture	Raising teenagers in a new culture	To encourage positive family relationships and discuss the challenges families may face when moving to Australia and ways in which these challenges can be overcome	• Parenting is challenging for everyone, regardless of where they come from	Group discussion and activities to show parents that they are not alone in the challenges that they face raising their teenagers, and as a way of sharing strategies for overcoming such challenges:
					• Changes/challenges faced in moving to a new country
					• Devising strategies for bridging the cultural gap between parents and teenagers to build more positive relationships.
				• Strategies to assist parents to be positive role models and reduce the cultural gap with their children	
					• Take home activity: discuss and implement the strategies for building positive families with their family they learned during the session, and make a plan to implement some of the strategies at home
3	Healthy lifestyles in a new culture	Body shape and active living	To promote physical activity as part of a healthy lifestyle and devise strategies to encourage regular physical activity	• Cultural differences regarding healthy body shape/size preferences	Group discussion and activities to examine various contributors to body shape and brainstorm strategies for increasing activity levels:
					• Selecting ‘healthy’ body shapes from the Australian and African perspective using silhouettes of various body shapes
					• Contributors to various body shapes/sizes
					• Differences in lifestyle between Africa and Australia
				• The benefits of physical activity for body weight, health, lifestyle, and families	
					• Types of sedentary activities, and strategies for increasing activity in the family
					• Take home activity: take note of the amount of electronic media the family uses in their leisure time and compare these to the recommended guidelines; and talk with their family about implementing strategies that will increase the health and activity levels of the family
				• Physical activity recommended guidelines	
4	Healthy lifestyles in a new culture	Understanding healthy eating	To promote the benefits of healthy eating and introduce the healthy food pyramid	• Basic principles of healthy eating using the ‘healthy food pyramid’ (adapted to include traditional African foods) as a guide, with a focus on individual foods/food groups and how often to consume those foods as part of a healthy and balanced diet	• Group discussion and activities to develop and consolidate understanding of the basic principles of healthy eating:
					• Discussion of the benefits of healthy eating
					• Estimating the number of teaspoons of sugar in glasses of common drinks
					• Sorting picture cards of foods into ‘eat most’, ‘eat moderately’, and ‘eat least’ (in accordance with the healthy food pyramid)
					• Take home activity: bring a copy of a favourite recipe that they cook frequently for a family meal. Put their (magnetic laminated) copy of the healthy food pyramid on their fridge or wall at home
5	Healthy families in a new culture	Improving family communication	To equip parents with tools (active listening, assertiveness, and “I” messages) for better communication with their family	• Cultural differences in family communication, and strategies for improving communication within the family	Group discussion and activities about strategies for good communication:
					• Discussion of challenges in communication with teenagers and brainstorming strategies for resolution of them
					• Activities to practice using active listening, assertiveness, and “I” messages using example scenarios which might be common African migrant families
					• Take home activity: practice the active listening and “I” message skills at home with their family
6	Healthy families in a new culture	Raising a family in Australia: discipline and conflict resolution	To equip parents with strategies to resolve conflict within their family and discuss cultural difference in discipline methods between Africa and Australia	• Different styles of parenting exist	Discussion and activities about resolving conflicts within the family:
				• Often Australian and African parents resolve family conflict	• Discussion of the similarities and differences in raising a family in Africa and Australia,
				• and discipline their children in different ways	
					• Brainstorming alternative solutions to discipline than corporal punishment
				• Australia has different laws compared to most African countries regarding discipline of children	
					• Steps for conflict resolution
					• Take home activity: practice the conflict resolution steps/alternate discipline strategies at home in family issues that arise
				• Alternative ways of disciplining children that are also helpful for building positive family relationships	
7	Healthy lifestyles in a new culture	Making healthy choices at home and away from home	To encourage parents to apply the principles of healthy eating principles when cooking meals at home and purchasing food outside of the home	• How to incorporate the principles of healthy eating when preparing meals at home and selecting foods away from home	Discussion and activities to develop skills in making healthier food choices for the family:
					• Modification of recipes to make them healthier
					• Sorting laminated A4 picture cards of various meals into ‘most healthy choice’, moderately healthy choice’ and ‘least healthy choice’
				• How to prevent food contamination and food poisoning (food safety)	
					• Food safety
					• Take home activity: test one of the modified recipes at home
8	Healthy lifestyles in a new culture	Healthy breakfasts, lunches and snacks	To encourage parents to plan meals and inspire appropriate, healthy meals for the whole family	• Small regular meals (e.g. 5–6 small meals per day) are recommended for good health	Discussion and activities to build skills in preparing healthy meals:
					• Discussion of the importance of breakfast
					• Brainstorming ideas to encourage breakfast and school lunch consumption
					• Discussion of ideas for healthy breakfast and lunches
					• Sorting laminated A4 picture cards of snacks into ‘healthy’ and ‘unhealthy’
				• Appropriate healthy foods for breakfast, lunches and snacks	
					• Developing a healthy meal plan (whole day) for their teenager using A4 laminated picture cards
				• Strategies for encouraging teenagers to eat their lunch at school	
					• Take home activity: discuss the session with their family and incorporate lessons learnt. And collect their grocery shopping receipts for 1 week to bring to session 9
9	Healthy lifestyles in a new culture	Smart shopping	To apply the principles of healthy to grocery shopping and discuss strategies for ‘smart shopping’ including budgeting, and reading food labels	• How to read food nutrition labels	Discussion and activities to build skills in shopping for healthy foods:
					• Analyse the healthfulness of their families food consumption using their grocery receipts and a colour code system
					• Practice reading food labels using A4 laminated picture cards and a laminated wallet-sized shopping tool
				• Pre-planning can help to make cost-effective and healthy food purchases	
					• Brainstorm ways to save money on food
					• Take home activity: devise solutions for making healthy changes to their grocery shopping with their family, plan their weekly family meals and prepare a shopping list, and practice reading nutrition labels using the shopping tool
				• Healthy eating is affordable and most of the time, healthy food is cheaper than unhealthy food	
					• Certificates handed out to participants who complete the program

### Program implementation

The sessions are designed to run in a group setting, as many of the program activities are discussion-based. Ideally, a group would have a minimum of six participants and up to 12 participants, to allow greater diversity in the group discussions. Though the session materials and program resources (refer to ‘Program resources and session materials’) are comprehensive, consistent with the cultural competence framework, the sessions should ideally be run by qualified bilingual health professionals such as dieticians or nurses, family support workers, and family counsellors who have experience working with African migrant families (or from the target communities for tailoring purpose). For instance, the ‘lifestyles’ module should be run by an Accredited Practising Dietician or nutritionist and the ‘families’ module should be run by a qualified social worker or parenting educator. Facilitators would be expected to become familiar with the program and program resources. If the facilitator is not multilingual in the languages of the program participants, a bilingual interpreter will be required at the sessions.

Several key resources have been developed to ensure that the HMFI is run consistently across differing groups and settings, and by different facilitators. The resources cover the following materials:

#### For participants and facilitators

1. ‘*Session PowerPoint Slides’* – one set of slides per session; to be projected onto a wall or screen whilst running the sessions to serve as a visual aid
2. *‘Activity materials’* – various materials to assist the participants in their learning, including:
  Picture cards of food, ingredients and relevant nutritional information – cards to facilitate discussion about the healthfulness of different foods and meals
  Session summaries – summary sheet of each session for the participants to take home and share with their families
  Other educational aids – healthy food pyramids adapted to include common traditional African foods for each participant to keep at home, and wallet-sized laminated food choice decision aids for use at the supermarket

#### For facilitators only

1. *‘Instruction Manual’* – a resource comprising an overview of the program and the relevant resources for running the program and a reference point for program implementation
2. *‘Facilitators Manual’* – the ‘go-to-guide’ for facilitators about how to conduct the HMFI program. It outlines the role and responsibilities of the facilitator, summarises each session, and provides instructions and guidance for facilitating the sessions and optimising discussion e.g. communication types, how to optimise discussion, how to avoid barriers to communication, how to use active listening, adult education principles, safety and ethical considerations (e.g. safety procedures, privacy and confidentiality)
3. ‘S*ession Guide’* – provides useful information for facilitators about how to run each session including discussion suggestions, activities, time for each session and activity, and background information regarding the facilitation materials.

## Discussion

This is the first study to develop a culturally competent obesity prevention program for sub-Saharan African migrant families in Australia. The developed program addresses two main components: healthy lifestyles and healthy families. The intervention is designed to be delivered to parents for three reasons: 1) to address the intergenerational acculturation gap, 2) to maximise the effect of parental involvement; and 3) to develop cultural competence strategies that maximise community ownership of the program.

Various studies have observed a dyadic acculturative stress resulting from differing pace of acculturation between migrant parents and their offspring, with children acculturating faster than their parents. The acculturation gap-distress hypothesis stipulates that when immigrant parents and children acculturate at different pace (e.g. inter-generational acculturation gap), the main outcomes include migrant parents and their children holding different values and preferences which often clash, family conflict as well as youth maladjustment [29,30]. It has been shown that improving parent–youth relationship quality helps youths cope more effectively with the stresses associated with adolescence and makes them responsive to positive parental influence related to a healthy lifestyle as well as a healthy body image and a healthy relationship with food [31-34]. In addition, evidence shows that parents and carers have a long-lasting impact on their child’s lifestyle behaviours throughout their life [35]. Parental support correlates with participation in physical activity, family commensality, food decision making, and family communication, while effective parenting and family cohesion offset vulnerability due to low socio-economic status (SES) [36]. Good parenting skills, positive family functioning, and higher parental control negate the deleterious effects of low SES on children [37]. Most importantly, there is emerging evidence suggesting that the success of family-based obesity prevention and treatment programs is greater when the focus is on training parents and maximising their involvement and influence at the household level; and when parents were the exclusive agents of change the results were superior to the conventional approach [38-40].

The intervention was developed in partnership with the community, and culturally competent strategies to sustain their participation and maximise their ownership of the program have been provided. Culturally tailored interventions should respect cultural practices, encourage retention of healthy traditional food practices, and work with people to identify ways by which they can modify their traditional foods to make manageable healthy changes. They should also identify and enlist influential family members, in particular those who shop for and prepare the food [41]. Both of those recommendations play a key role in the HMFI program – parents, as the food “gatekeepers” within the family are encouraged to attend the program sessions, and the program sessions will cater to different levels of acculturation by focusing upon Australian and traditional African foods. The proposed model is consistent with best practice in community partnership and how it relates to public health [42].

There are some limitations worth outlining. The paper provides a description of a best practice approach to developing interventions that would be culturally relevant and acceptable to immigrant and other ethnic minority groups. The described process adopted a culturally competence framework and would be well-suited to immigrant or other ethnic minority groups when researchers and practitioners are interested in developing culturally competent programs. Nevertheless, the paper is not about the evaluation of the program as we do not yet have data on the program implementation, tailoring process, and program efficacy. A randomised controlled trial is planned in the near future to evaluate the intervention’s impact on primary outcome (child body mass index) and secondary outcomes (parenting style, family functioning, food habits, level of physical activity). The evaluation will also shed light on the steps required to tailor the intervention to other culturally and linguistically diverse populations (e.g. evaluating implementation-related factors such as considerations that need to be made before and during implementation of the program, things that work/ don’t work for mobilising participants, keeping participants engaged in the sessions, and how the program sessions should be delivered in a culturally appropriate way).

## Conclusion

The Healthy Migrant Families Initiative developed in this project is evidence-based and grounded in cultural engagement principles. Implementation and evaluation using the same cultural engagement principles will further add to interventions to promote culturally competent obesity prevention strategies for African and other communities settling in Australia.
